# Short- and Long-Term Consequences of CO_2_ Pneumoperitoneum Impact on Children’s Health

**DOI:** 10.5152/TJAR.2021.21193

**Published:** 2022-12-01

**Authors:** Amirkhan K. Baimaganbetov, Michael Stark, Andrea Tinelli, Ospan A. Mynbaev

**Affiliations:** 1 Department of Traumatology, Orthopaedics and Oncology, Khoja-Akhmet Yassawi International Kazakh-Turkish University, Turkestan Region, Kazakhstan; 2 Headquarters New European Surgical Academy, Berlin, Germany; 3 Department of Obstetrics & Gynecology, Veris delli Ponti Hospital, Italy; 4 Laboratory of Human Physiology Moscow Institute of Physics and Technology, National Research Institute, Moscow, Russia

We read with great interest the article by Kılınç et al^1^ and we congratulate the authors for a well-designed prospective, observational study. The authors perioperatively monitored the regional splanchnic saturation (rSPcO_2_), regional cerebral oxygen saturation (rScO_2_), peripheral oxygen saturation (SpO_2_), end-tidal CO_2_ (Et-CO_2_), heart rate, mean arterial pressure, and CO_2_ insufflation pressure (IP) during laparoscopic surgery in children. An increased Et-CO_2_ was associated with decreased regional oxygen saturation parameters (rSPcO_2_ and rScO_2_) during laparoscopic surgery with CO_2_ pneumoperitoneum. We also observed analogously increased Et-CO_2_ in children during laparoscopic surgery^2^ associated with slight blood gas values and acid–base balance changes, associated with decreased skin temperature and urine output. We also found increased concentrations of pCO_2_ in the carotid venous and peripheral ear arterial blood with increased venous-to-arterial differences of CO_2_ tension with lower pH and tissue acidosis during CO_2_ pneumoperitoneum in the rabbit model.^3^ These experimental results are certainly meaningful for paediatric surgery as there is a correlation among this model to physiological changes in children and therefore decreased regional oxygen saturation might be associated with increased CO_2_ concentrations with lower pH and tissue acidosis in these regions. We believe that such decreased regional oxygen saturation in different locations is an important outcome of this study,^1^ and all these changes are the only short-term impact of CO_2_ pneumoperitoneum. Possible future consequences of decreased cerebral oxygen saturation and tissue acidosis might influence postoperative cognitive function.^4^ Risk factors of this condition in relation to laparoscopic surgery and general anaesthesia have been extensively studied.^5-7^ Short-term changes related to CO_2_ pneumoperitoneum during laparoscopic surgery are well documented these days although its long-term consequences remaining unknown. Children and adults are vulnerable to post-surgical complications. It is very difficult to learn the postoperative cognitive function in paediatric patients; therefore, further observations of children after such surgical treatment are needed to study the long-term consequences of CO_2_ pneumoperitoneum and general anaesthesia.
